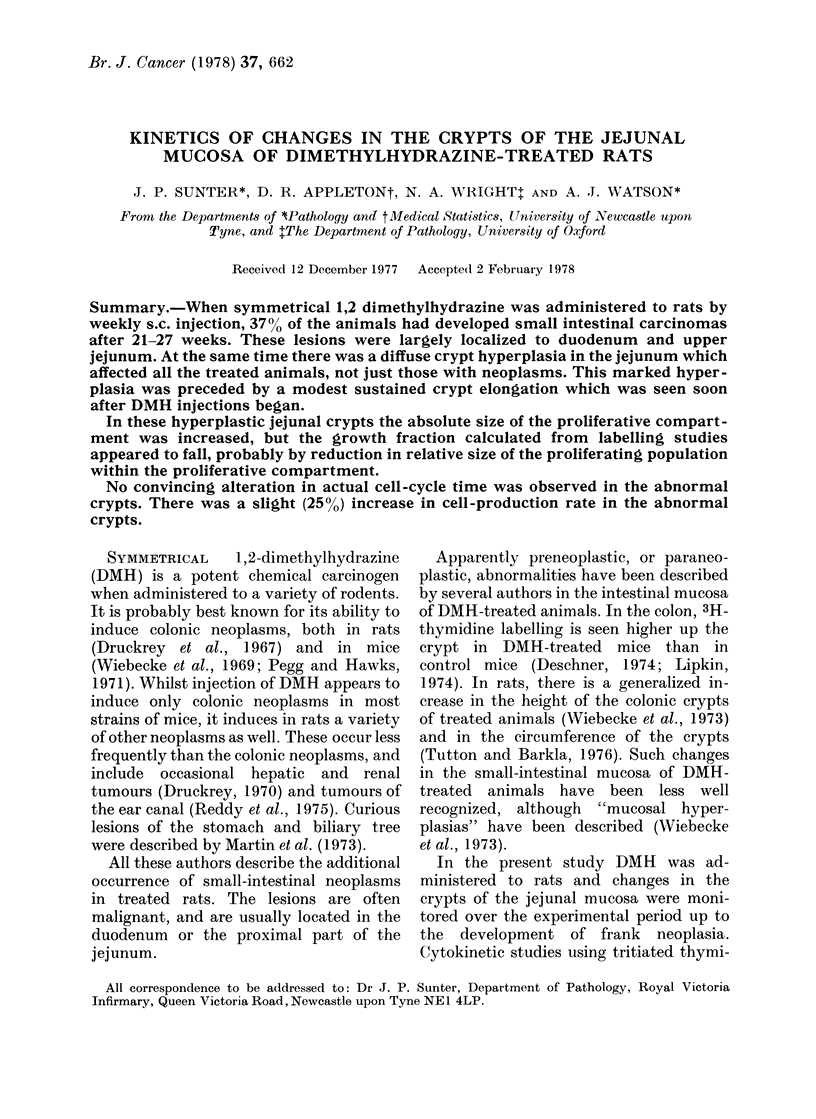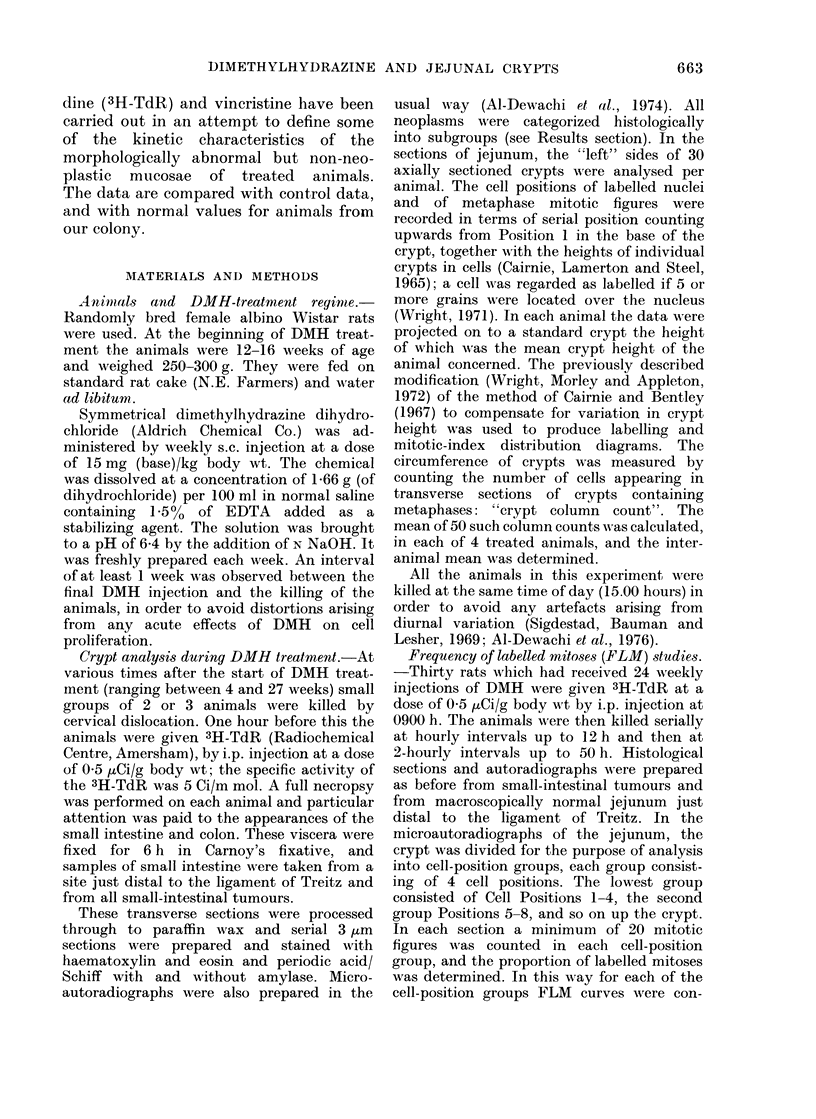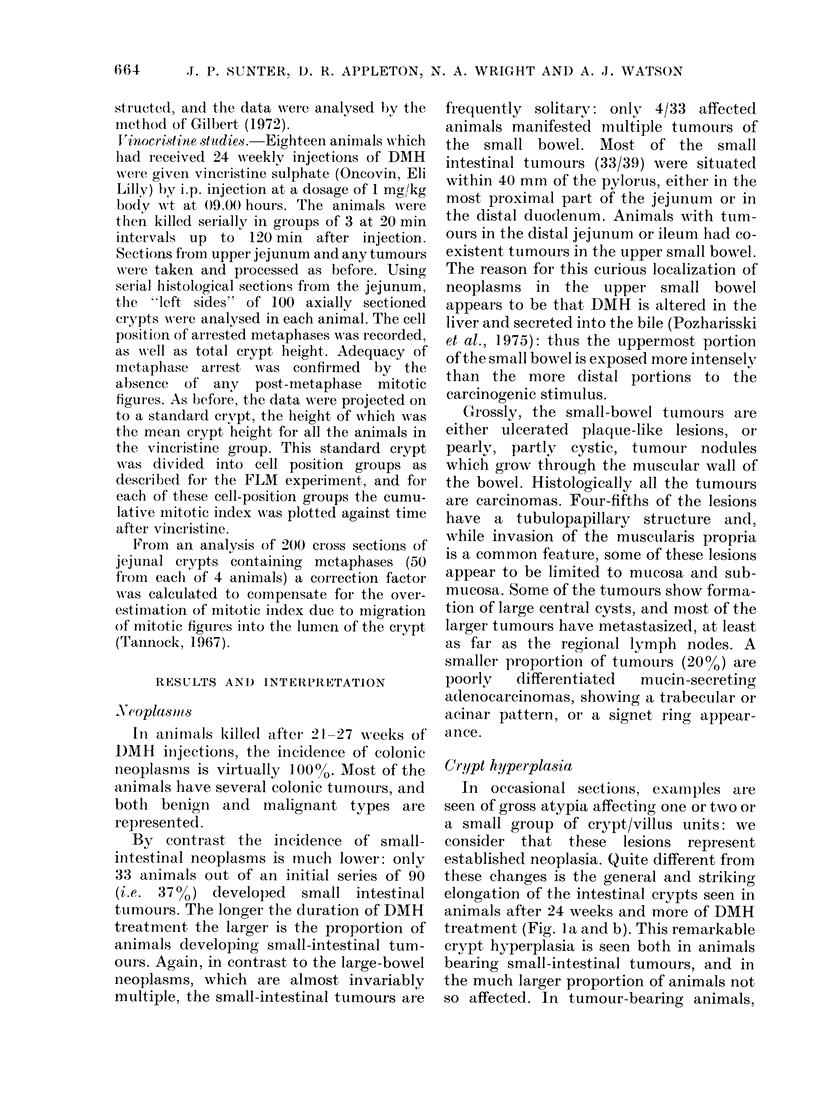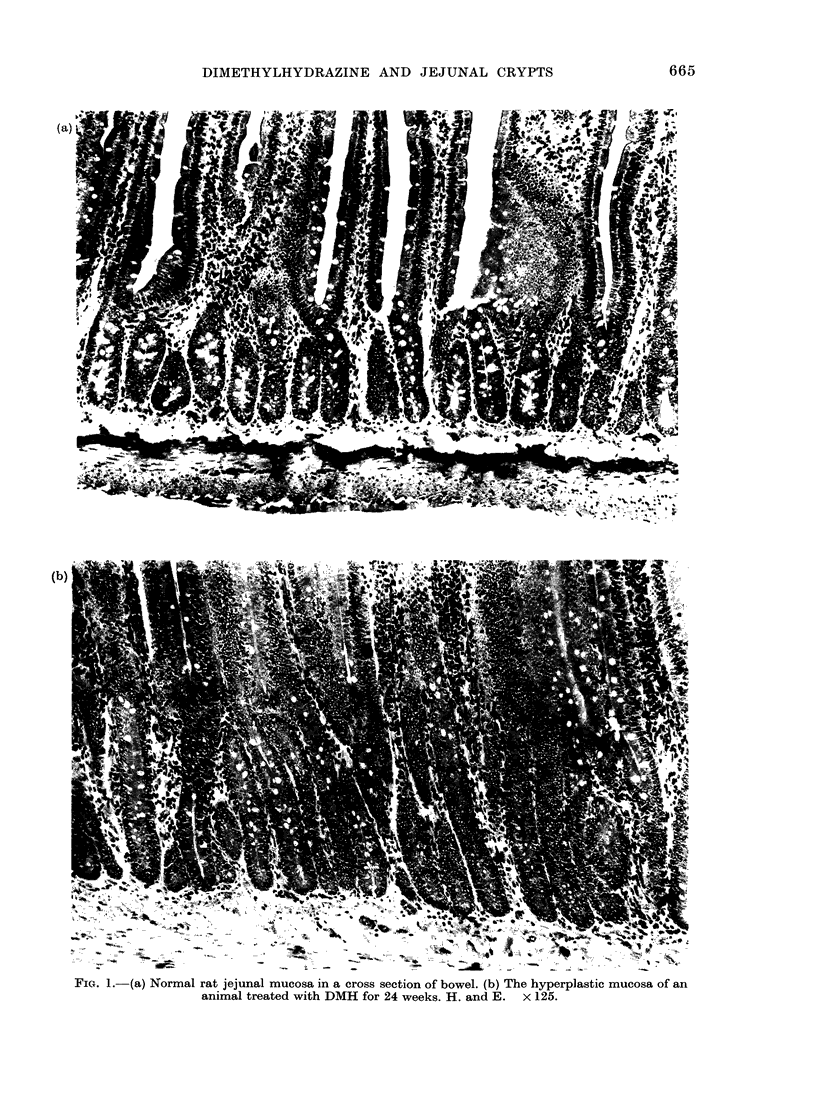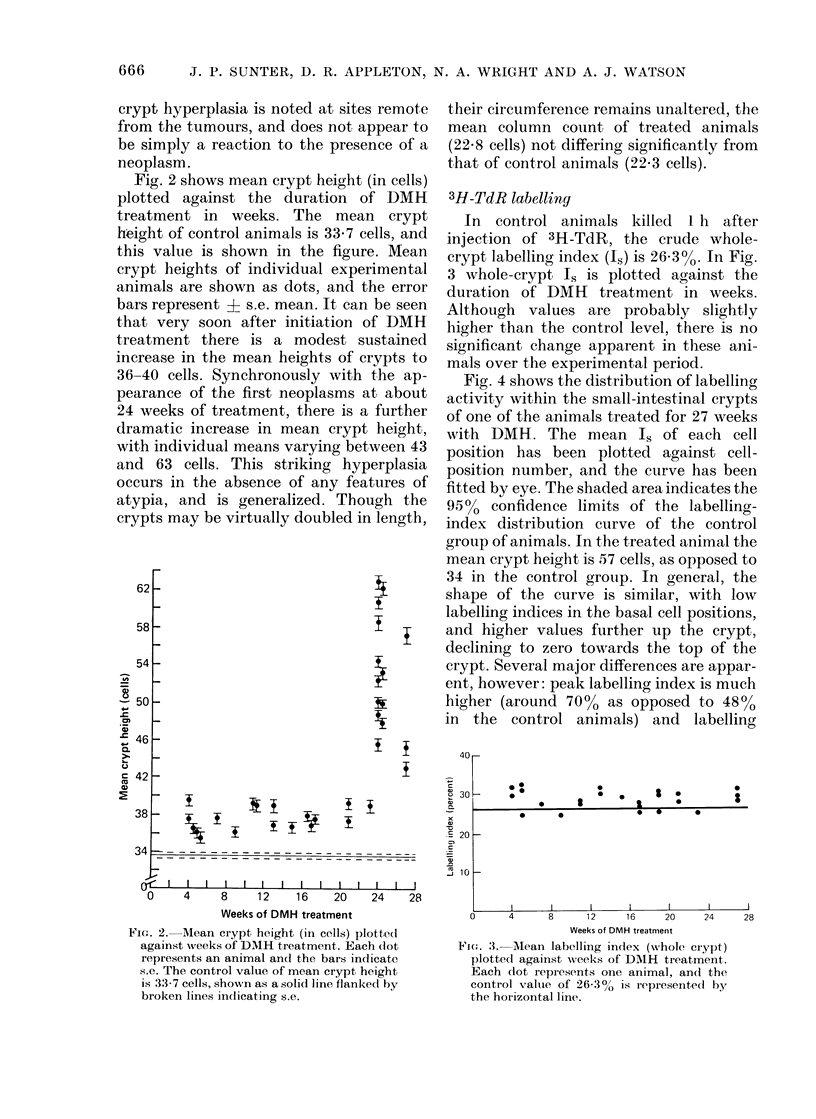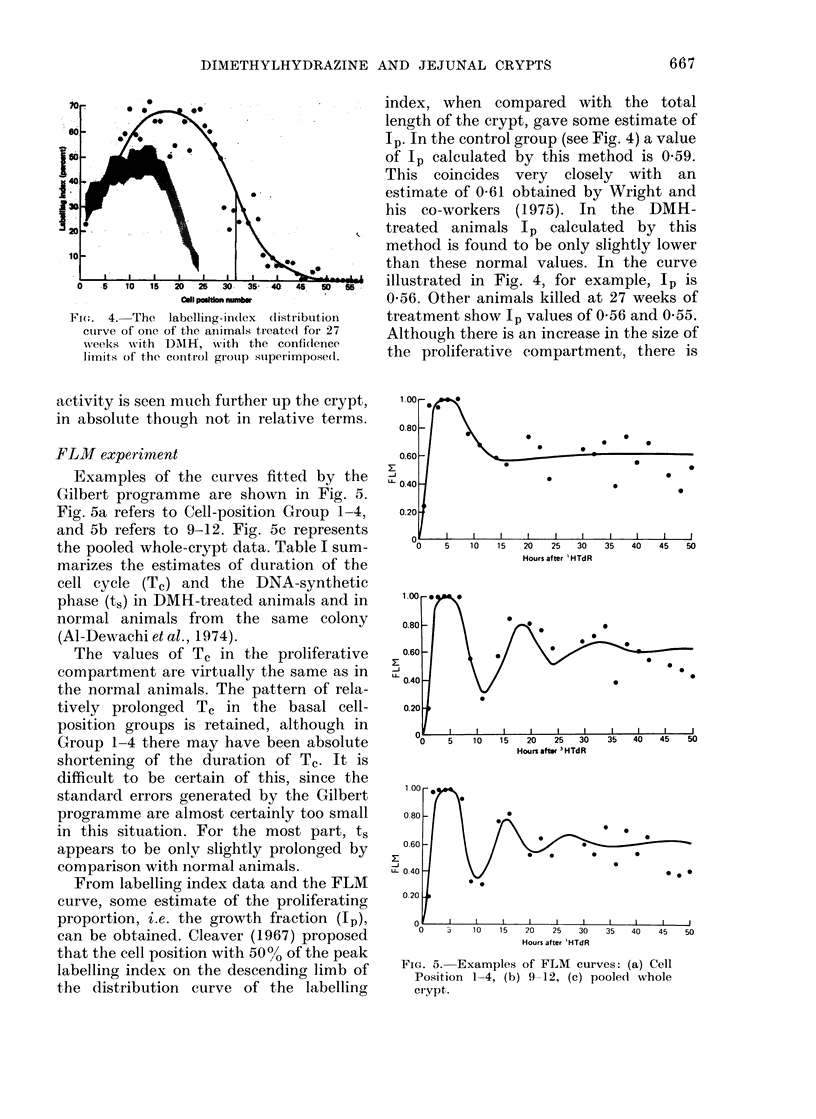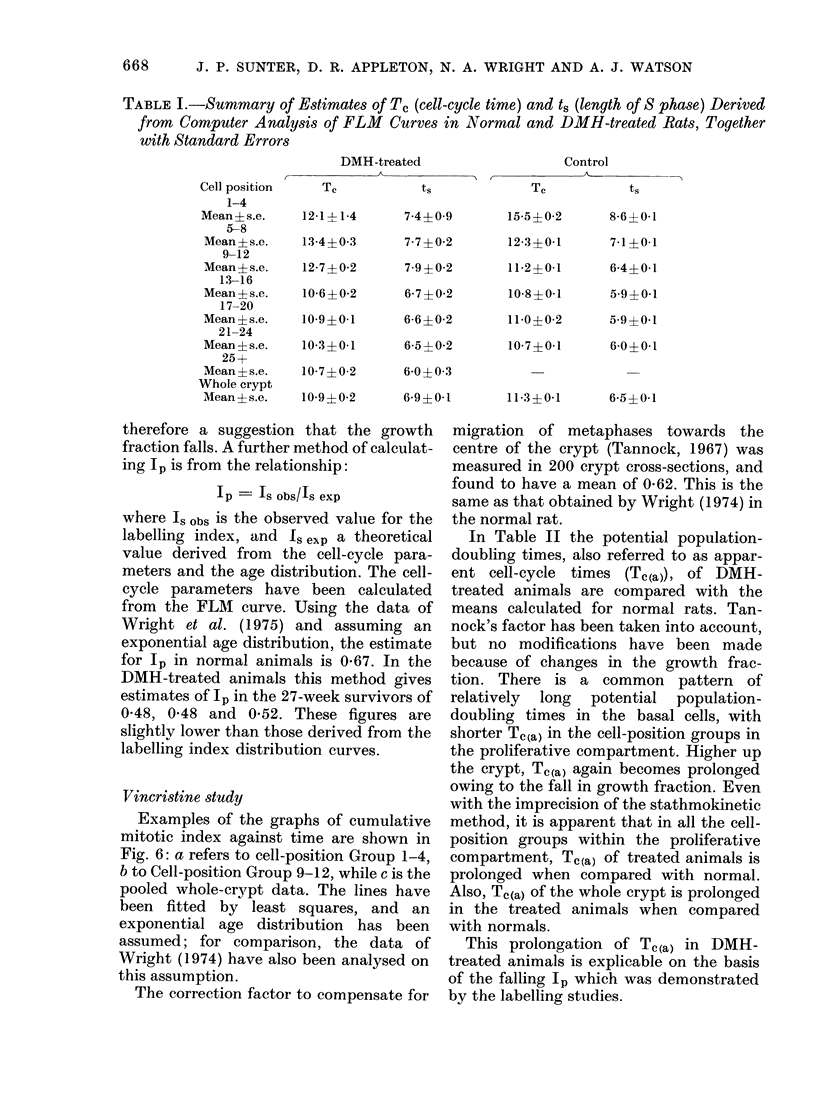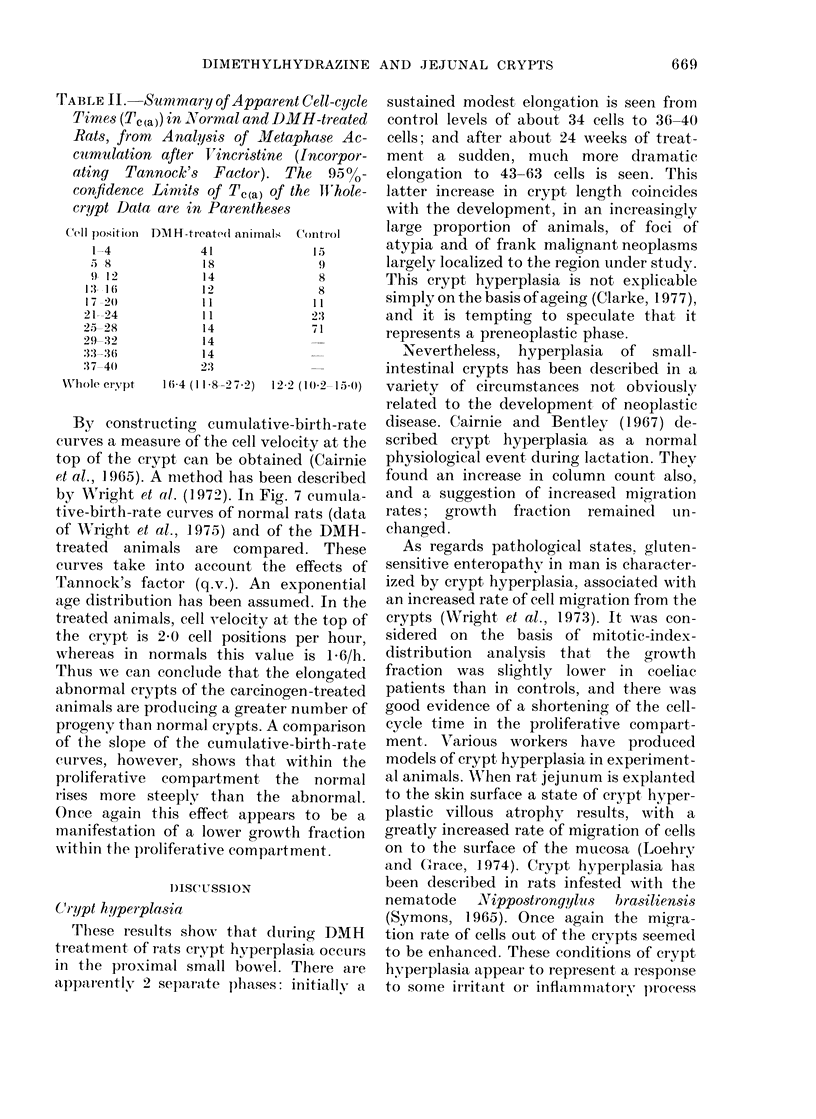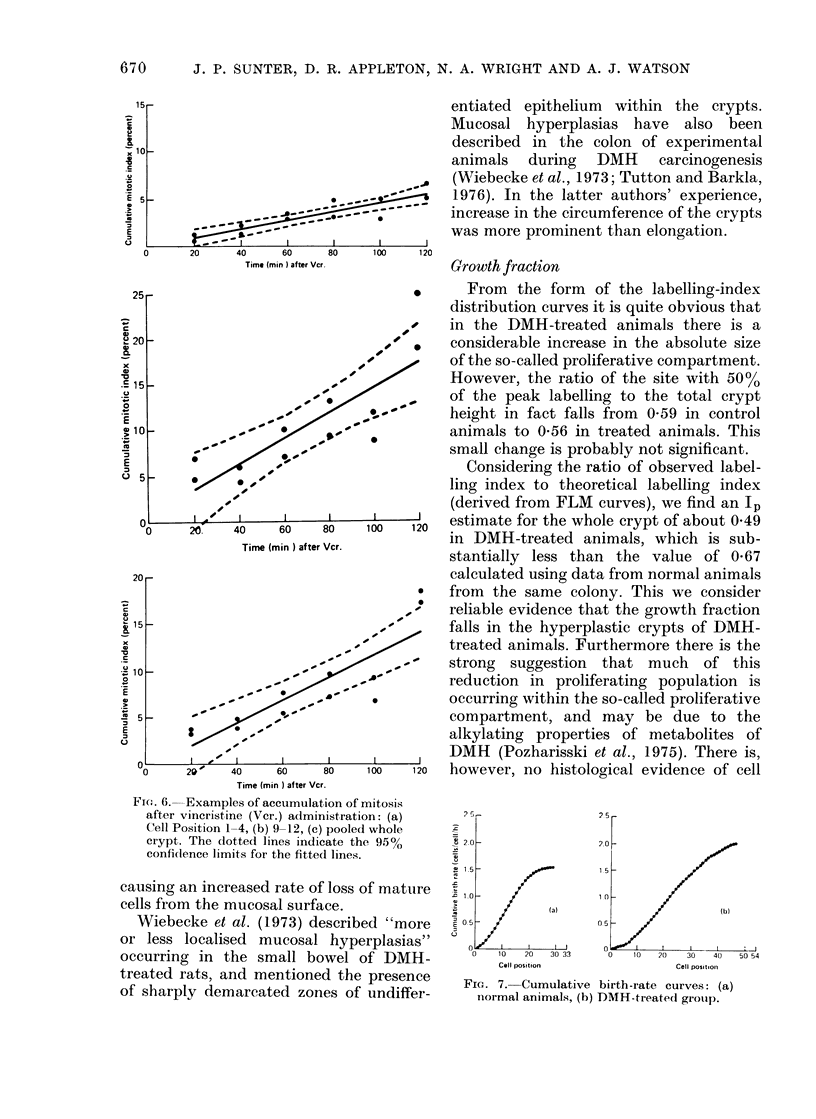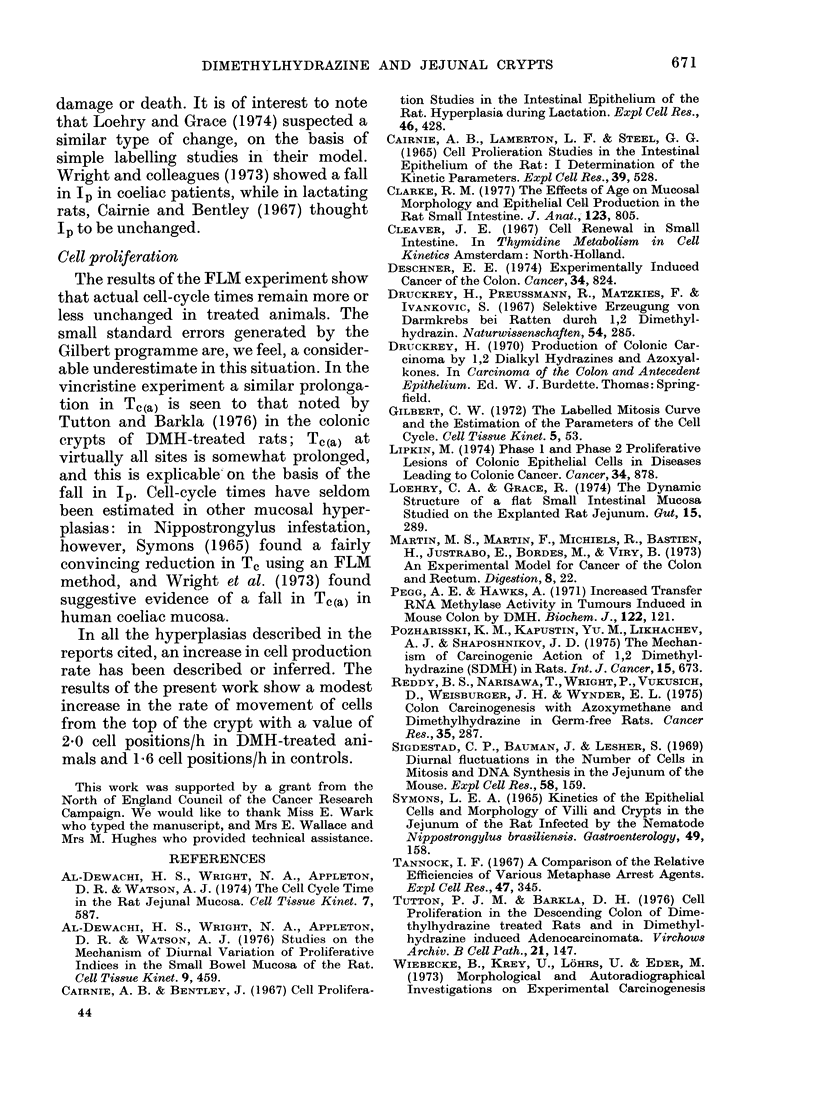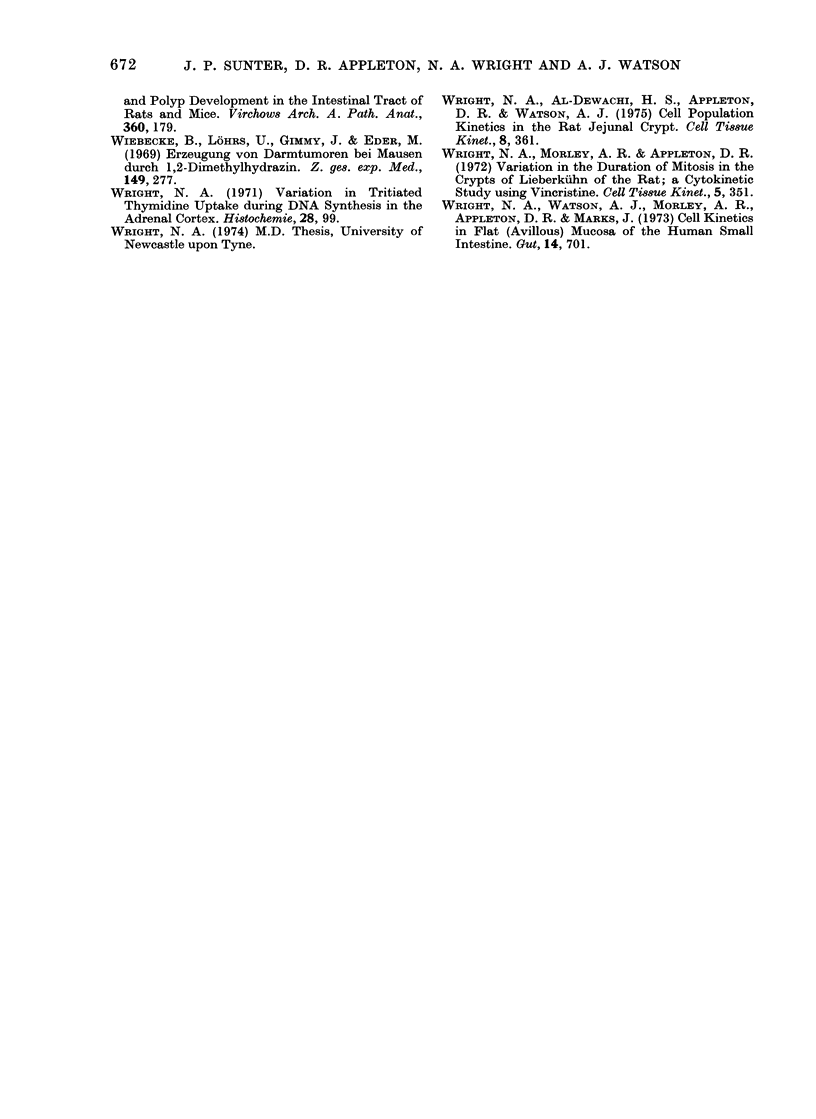# Kinetics of changes in the crypts of the jejunal mucosa of dimethylhydrazine-treated rats.

**DOI:** 10.1038/bjc.1978.104

**Published:** 1978-05

**Authors:** J. P. Sunter, D. R. Appleton, N. A. Wright, A. J. Watson

## Abstract

**Images:**


*Br. J. Cancer* (1978) **37**, 662

### KINETICS OF CHANGES IN THE CRYPTS OF THE JEJUNAL MUCOSA OF DIMETHYLHYDRAZINE-TREATED RATS

J. P. SUNTER*, D. R. APPLETON†, N. A. WRIGHT‡ AND A. J. WATSON*

*From the Departments of *Pathology and †Medical Statistics, University of Newcastle upon Tyne, and ‡The Department of Pathology, University of Oxford*

Received 12 December 1977 Accepted 2 February 1978

**Summary.—When symmetrical 1,2 dimethylhydrazine was administered to rats by weekly s.c. injection, 37% of the animals had developed small intestinal carcinomas after 21–27 weeks. These lesions were largely localized to duodenum and upper jejunum. At the same time there was a diffuse crypt hyperplasia in the jejunum which affected all the treated animals, not just those with neoplasms. This marked hyperplasia was preceded by a modest sustained crypt elongation which was seen soon after DMH injections began.**

**In these hyperplastic jejunal crypts the absolute size of the proliferative compartment was increased, but the growth fraction calculated from labelling studies appeared to fall, probably by reduction in relative size of the proliferating population within the proliferative compartment.**

**No convincing alteration in actual cell-cycle time was observed in the abnormal crypts. There was a slight (25%) increase in cell-production rate in the abnormal crypts.**

SYMMETRICAL 1,2-dimethylhydrazine (DMH) is a potent chemical carcinogen when administered to a variety of rodents. It is probably best known for its ability to induce colonic neoplasms, both in rats (Druckrey *et al.*, 1967) and in mice (Wiebecke *et al.*, 1969; Pegg and Hawks, 1971). Whilst injection of DMH appears to induce only colonic neoplasms in most strains of mice, it induces in rats a variety of other neoplasms as well. These occur less frequently than the colonic neoplasms, and include occasional hepatic and renal tumours (Druckrey, 1970) and tumours of the ear canal (Reddy *et al.*, 1975). Curious lesions of the stomach and biliary tree were described by Martin *et al.* (1973).

All these authors describe the additional occurrence of small-intestinal neoplasms in treated rats. The lesions are often malignant, and are usually located in the duodenum or the proximal part of the jejunum.

Apparently preneoplastic, or paraneoplastic, abnormalities have been described by several authors in the intestinal mucosa of DMH-treated animals. In the colon, $^{3}$H-thymidine labelling is seen higher up the crypt in DMH-treated mice than in control mice (Deschner, 1974; Lipkin, 1974). In rats, there is a generalized increase in the height of the colonic crypts of treated animals (Wiebecke *et al.*, 1973) and in the circumference of the crypts (Tutton and Barkla, 1976). Such changes in the small-intestinal mucosa of DMH-treated animals have been less well recognized, although "mucosal hyperplasias" have been described (Wiebecke *et al.*, 1973).

In the present study DMH was administered to rats and changes in the crypts of the jejunal mucosa were monitored over the experimental period up to the development of frank neoplasia. Cytokinetic studies using tritiated thymi-

All correspondence to be addressed to: Dr J. P. Sunter, Department of Pathology, Royal Victoria Infirmary, Queen Victoria Road, Newcastle upon Tyne NE1 4LP.

dine ($^3$H-TdR) and vincristine have been carried out in an attempt to define some of the kinetic characteristics of the morphologically abnormal but non-neoplastic mucosae of treated animals. The data are compared with control data, and with normal values for animals from our colony.

### MATERIALS AND METHODS

*Animals and DMH-treatment regime.*—Randomly bred female albino Wistar rats were used. At the beginning of DMH treatment the animals were 12–16 weeks of age and weighed 250–300 g. They were fed on standard rat cake (N.E. Farmers) and water *ad libitum.*

Symmetrical dimethylhydrazine dihydrochloride (Aldrich Chemical Co.) was administered by weekly s.c. injection at a dose of 15 mg (base)/kg body wt. The chemical was dissolved at a concentration of 1·66 g (of dihydrochloride) per 100 ml in normal saline containing 1·5% of EDTA added as a stabilizing agent. The solution was brought to a pH of 6·4 by the addition of N NaOH. It was freshly prepared each week. An interval of at least 1 week was observed between the final DMH injection and the killing of the animals, in order to avoid distortions arising from any acute effects of DMH on cell proliferation.

*Crypt analysis during DMH treatment.*—At various times after the start of DMH treatment (ranging between 4 and 27 weeks) small groups of 2 or 3 animals were killed by cervical dislocation. One hour before this the animals were given $^3$H-TdR (Radiochemical Centre, Amersham), by i.p. injection at a dose of 0·5 μCi/g body wt; the specific activity of the $^3$H-TdR was 5 Ci/m mol. A full necropsy was performed on each animal and particular attention was paid to the appearances of the small intestine and colon. These viscera were fixed for 6 h in Carnoy's fixative, and samples of small intestine were taken from a site just distal to the ligament of Treitz and from all small-intestinal tumours.

These transverse sections were processed through to paraffin wax and serial 3 μm sections were prepared and stained with haematoxylin and eosin and periodic acid/Schiff with and without amylase. Microautoradiographs were also prepared in the usual way (Al-Dewachi *et al.*, 1974). All neoplasms were categorized histologically into subgroups (see Results section). In the sections of jejunum, the "left" sides of 30 axially sectioned crypts were analysed per animal. The cell positions of labelled nuclei and of metaphase mitotic figures were recorded in terms of serial position counting upwards from Position 1 in the base of the crypt, together with the heights of individual crypts in cells (Cairnie, Lamerton and Steel, 1965); a cell was regarded as labelled if 5 or more grains were located over the nucleus (Wright, 1971). In each animal the data were projected on to a standard crypt the height of which was the mean crypt height of the animal concerned. The previously described modification (Wright, Morley and Appleton, 1972) of the method of Cairnie and Bentley (1967) to compensate for variation in crypt height was used to produce labelling and mitotic-index distribution diagrams. The circumference of crypts was measured by counting the number of cells appearing in transverse sections of crypts containing metaphases: "crypt column count". The mean of 50 such column counts was calculated, in each of 4 treated animals, and the inter-animal mean was determined.

All the animals in this experiment were killed at the same time of day (15.00 hours) in order to avoid any artefacts arising from diurnal variation (Sigdestad, Bauman and Lesher, 1969; Al-Dewachi *et al.*, 1976).

*Frequency of labelled mitoses (FLM) studies.*—Thirty rats which had received 24 weekly injections of DMH were given $^3$H-TdR at a dose of 0·5 μCi/g body wt by i.p. injection at 0900 h. The animals were then killed serially at hourly intervals up to 12 h and then at 2-hourly intervals up to 50 h. Histological sections and autoradiographs were prepared as before from small-intestinal tumours and from macroscopically normal jejunum just distal to the ligament of Treitz. In the microautoradiographs of the jejunum, the crypt was divided for the purpose of analysis into cell-position groups, each group consisting of 4 cell positions. The lowest group consisted of Cell Positions 1–4, the second group Positions 5–8, and so on up the crypt. In each section a minimum of 20 mitotic figures was counted in each cell-position group, and the proportion of labelled mitoses was determined. In this way for each of the cell-position groups FLM curves were constructed, and the data were analysed by the method of Gilbert (1972).

*Vinocristine studies.*—Eighteen animals which had received 24 weekly injections of DMH were given vincristine sulphate (Oncovin, Eli Lilly) by i.p. injection at a dosage of 1 mg/kg body wt at 09.00 hours. The animals were then killed serially in groups of 3 at 20 min intervals up to 120 min after injection. Sections from upper jejunum and any tumours were taken and processed as before. Using serial histological sections from the jejunum, the "left sides" of 100 axially sectioned crypts were analysed in each animal. The cell position of arrested metaphases was recorded, as well as total crypt height. Adequacy of metaphase arrest was confirmed by the absence of any post-metaphase mitotic figures. As before, the data were projected on to a standard crypt, the height of which was the mean crypt height for all the animals in the vincristine group. This standard crypt was divided into cell position groups as described for the FLM experiment, and for each of these cell-position groups the cumulative mitotic index was plotted against time after vincristine.

From an analysis of 200 cross sections of jejunal crypts containing metaphases (50 from each of 4 animals) a correction factor was calculated to compensate for the over-estimation of mitotic index due to migration of mitotic figures into the lumen of the crypt (Tannock, 1967).

### RESULTS AND INTERPRETATION

### *Neoplasms*

In animals killed after 21–27 weeks of DMH injections, the incidence of colonic neoplasms is virtually 100%. Most of the animals have several colonic tumours, and both benign and malignant types are represented.

By contrast the incidence of small-intestinal neoplasms is much lower: only 33 animals out of an initial series of 90 (*i.e.* 37%) developed small intestinal tumours. The longer the duration of DMH treatment the larger is the proportion of animals developing small-intestinal tumours. Again, in contrast to the large-bowel neoplasms, which are almost invariably multiple, the small-intestinal tumours are frequently solitary: only 4/33 affected animals manifested multiple tumours of the small bowel. Most of the small intestinal tumours (33/39) were situated within 40 mm of the pylorus, either in the most proximal part of the jejunum or in the distal duodenum. Animals with tumours in the distal jejunum or ileum had co-existent tumours in the upper small bowel. The reason for this curious localization of neoplasms in the upper small bowel appears to be that DMH is altered in the liver and secreted into the bile (Pozharisski *et al.*, 1975): thus the uppermost portion of the small bowel is exposed more intensely than the more distal portions to the carcinogenic stimulus.

Grossly, the small-bowel tumours are either ulcerated plaque-like lesions, or pearly, partly cystic, tumour nodules which grow through the muscular wall of the bowel. Histologically all the tumours are carcinomas. Four-fifths of the lesions have a tubulopapillary structure and, while invasion of the muscularis propria is a common feature, some of these lesions appear to be limited to mucosa and sub-mucosa. Some of the tumours show formation of large central cysts, and most of the larger tumours have metastasized, at least as far as the regional lymph nodes. A smaller proportion of tumours (20%) are poorly differentiated mucin-secreting adenocarcinomas, showing a trabecular or acinar pattern, or a signet ring appearance.

### *Crypt hyperplasia*

In occasional sections, examples are seen of gross atypia affecting one or two or a small group of crypt/villus units: we consider that these lesions represent established neoplasia. Quite different from these changes is the general and striking elongation of the intestinal crypts seen in animals after 24 weeks and more of DMH treatment (Fig. 1a and b). This remarkable crypt hyperplasia is seen both in animals bearing small-intestinal tumours, and in the much larger proportion of animals not so affected. In tumour-bearing animals,

FIG. 1.—(a) Normal rat jejunal mucosa in a cross section of bowel. (b) The hyperplastic mucosa of an animal treated with DMH for 24 weeks. H. and E. ×125.

crypt hyperplasia is noted at sites remote from the tumours, and does not appear to be simply a reaction to the presence of a neoplasm.

Fig. 2 shows mean crypt height (in cells) plotted against the duration of DMH treatment in weeks. The mean crypt height of control animals is 33·7 cells, and this value is shown in the figure. Mean crypt heights of individual experimental animals are shown as dots, and the error bars represent ± s.e. mean. It can be seen that very soon after initiation of DMH treatment there is a modest sustained increase in the mean heights of crypts to 36–40 cells. Synchronously with the appearance of the first neoplasms at about 24 weeks of treatment, there is a further dramatic increase in mean crypt height, with individual means varying between 43 and 63 cells. This striking hyperplasia occurs in the absence of any features of atypia, and is generalized. Though the crypts may be virtually doubled in length, their circumference remains unaltered, the mean column count of treated animals (22·8 cells) not differing significantly from that of control animals (22·3 cells).

Fig. 2.—Mean crypt height (in cells) plotted against weeks of DMH treatment. Each dot represents an animal and the bars indicate s.e. The control value of mean crypt height is 33·7 cells, shown as a solid line flanked by broken lines indicating s.e.

### ${}^3$H-TdR labelling

In control animals killed 1 h after injection of ${}^3$H-TdR, the crude whole-crypt labelling index ($I_S$) is 26·3%. In Fig. 3 whole-crypt $I_S$ is plotted against the duration of DMH treatment in weeks. Although values are probably slightly higher than the control level, there is no significant change apparent in these animals over the experimental period.

Fig. 4 shows the distribution of labelling activity within the small-intestinal crypts of one of the animals treated for 27 weeks with DMH. The mean $I_S$ of each cell position has been plotted against cell-position number, and the curve has been fitted by eye. The shaded area indicates the 95% confidence limits of the labelling-index distribution curve of the control group of animals. In the treated animal the mean crypt height is 57 cells, as opposed to 34 in the control group. In general, the shape of the curve is similar, with low labelling indices in the basal cell positions, and higher values further up the crypt, declining to zero towards the top of the crypt. Several major differences are apparent, however: peak labelling index is much higher (around 70% as opposed to 48% in the control animals) and labelling

Fig. 3.—Mean labelling index (whole crypt) plotted against weeks of DMH treatment. Each dot represents one animal, and the control value of 26·3% is represented by the horizontal line.

FIG. 4.—The labelling-index distribution curve of one of the animals treated for 27 weeks with DMH, with the confidence limits of the control group superimposed.

index, when compared with the total length of the crypt, gave some estimate of $I_p$. In the control group (see Fig. 4) a value of $I_p$ calculated by this method is 0·59. This coincides very closely with an estimate of 0·61 obtained by Wright and his co-workers (1975). In the DMH-treated animals $I_p$ calculated by this method is found to be only slightly lower than these normal values. In the curve illustrated in Fig. 4, for example, $I_p$ is 0·56. Other animals killed at 27 weeks of treatment show $I_p$ values of 0·56 and 0·55. Although there is an increase in the size of the proliferative compartment, there is activity is seen much further up the crypt, in absolute though not in relative terms.

*FLM experiment*

Examples of the curves fitted by the Gilbert programme are shown in Fig. 5. Fig. 5a refers to Cell-position Group 1–4, and 5b refers to 9–12. Fig. 5c represents the pooled whole-crypt data. Table I summarizes the estimates of duration of the cell cycle ($T_c$) and the DNA-synthetic phase ($t_s$) in DMH-treated animals and in normal animals from the same colony (Al-Dewachi *et al.*, 1974).

The values of $T_c$ in the proliferative compartment are virtually the same as in the normal animals. The pattern of relatively prolonged $T_c$ in the basal cell-position groups is retained, although in Group 1–4 there may have been absolute shortening of the duration of $T_c$. It is difficult to be certain of this, since the standard errors generated by the Gilbert programme are almost certainly too small in this situation. For the most part, $t_s$ appears to be only slightly prolonged by comparison with normal animals.

From labelling index data and the FLM curve, some estimate of the proliferating proportion, *i.e.* the growth fraction ($I_p$), can be obtained. Cleaver (1967) proposed that the cell position with 50% of the peak labelling index on the descending limb of the distribution curve of the labelling

FIG. 5.—Examples of FLM curves: (a) Cell Position 1–4, (b) 9–12, (c) pooled whole crypt.

TABLE I.—*Summary of Estimates of $T_C$ (cell-cycle time) and $t_S$ (length of S phase) Derived from Computer Analysis of FLM Curves in Normal and DMH-treated Rats, Together with Standard Errors*

| Cell position | DMH-treated $T_C$ | DMH-treated $t_S$ | Control $T_C$ | Control $t_S$ |
|---|---|---|---|---|
| 1–4 Mean ± s.e. | 12·1 ± 1·4 | 7·4 ± 0·9 | 15·5 ± 0·2 | 8·6 ± 0·1 |
| 5–8 Mean ± s.e. | 13·4 ± 0·3 | 7·7 ± 0·2 | 12·3 ± 0·1 | 7·1 ± 0·1 |
| 9–12 Mean ± s.e. | 12·7 ± 0·2 | 7·9 ± 0·2 | 11·2 ± 0·1 | 6·4 ± 0·1 |
| 13–16 Mean ± s.e. | 10·6 ± 0·2 | 6·7 ± 0·2 | 10·8 ± 0·1 | 5·9 ± 0·1 |
| 17–20 Mean ± s.e. | 10·9 ± 0·1 | 6·6 ± 0·2 | 11·0 ± 0·2 | 5·9 ± 0·1 |
| 21–24 Mean ± s.e. | 10·3 ± 0·1 | 6·5 ± 0·2 | 10·7 ± 0·1 | 6·0 ± 0·1 |
| 25+ Mean ± s.e. | 10·7 ± 0·2 | 6·0 ± 0·3 | — | — |
| Whole crypt Mean ± s.e. | 10·9 ± 0·2 | 6·9 ± 0·1 | 11·3 ± 0·1 | 6·5 ± 0·1 |

therefore a suggestion that the growth fraction falls. A further method of calculating $I_P$ is from the relationship:

$$I_P = I_{S\ obs}/I_{S\ exp}$$

where $I_{S\ obs}$ is the observed value for the labelling index, and $I_{S\ exp}$ a theoretical value derived from the cell-cycle parameters and the age distribution. The cell-cycle parameters have been calculated from the FLM curve. Using the data of Wright *et al.* (1975) and assuming an exponential age distribution, the estimate for $I_P$ in normal animals is 0·67. In the DMH-treated animals this method gives estimates of $I_P$ in the 27-week survivors of 0·48, 0·48 and 0·52. These figures are slightly lower than those derived from the labelling index distribution curves.

### *Vincristine study*

Examples of the graphs of cumulative mitotic index against time are shown in Fig. 6: *a* refers to cell-position Group 1–4, *b* to Cell-position Group 9–12, while *c* is the pooled whole-crypt data. The lines have been fitted by least squares, and an exponential age distribution has been assumed; for comparison, the data of Wright (1974) have also been analysed on this assumption.

The correction factor to compensate for migration of metaphases towards the centre of the crypt (Tannock, 1967) was measured in 200 crypt cross-sections, and found to have a mean of 0·62. This is the same as that obtained by Wright (1974) in the normal rat.

In Table II the potential population-doubling times, also referred to as apparent cell-cycle times ($T_{C(a)}$), of DMH-treated animals are compared with the means calculated for normal rats. Tannock's factor has been taken into account, but no modifications have been made because of changes in the growth fraction. There is a common pattern of relatively long potential population-doubling times in the basal cells, with shorter $T_{C(a)}$ in the cell-position groups in the proliferative compartment. Higher up the crypt, $T_{C(a)}$ again becomes prolonged owing to the fall in growth fraction. Even with the imprecision of the stathmokinetic method, it is apparent that in all the cell-position groups within the proliferative compartment, $T_{C(a)}$ of treated animals is prolonged when compared with normal. Also, $T_{C(a)}$ of the whole crypt is prolonged in the treated animals when compared with normals.

This prolongation of $T_{C(a)}$ in DMH-treated animals is explicable on the basis of the falling $I_P$ which was demonstrated by the labelling studies.

TABLE II.—*Summary of Apparent Cell-cycle Times ($T_{c(a)}$) in Normal and DMH-treated Rats, from Analysis of Metaphase Accumulation after Vincristine (Incorporating Tannock's Factor). The 95%-confidence Limits of $T_{c(a)}$ of the Whole-crypt Data are in Parentheses*

| Cell position | DMH-treated animals | Control |
|---|---|---|
| 1–4 | 41 | 15 |
| 5–8 | 18 | 9 |
| 9–12 | 14 | 8 |
| 13–16 | 12 | 8 |
| 17–20 | 11 | 11 |
| 21–24 | 11 | 23 |
| 25–28 | 14 | 71 |
| 29–32 | 14 | — |
| 33–36 | 14 | — |
| 37–40 | 23 | — |
| Whole crypt | 16·4 (11·8–27·2) | 12·2 (10·2–15·0) |

By constructing cumulative-birth-rate curves a measure of the cell velocity at the top of the crypt can be obtained (Cairnie *et al.*, 1965). A method has been described by Wright *et al.* (1972). In Fig. 7 cumulative-birth-rate curves of normal rats (data of Wright *et al.*, 1975) and of the DMH-treated animals are compared. These curves take into account the effects of Tannock's factor (q.v.). An exponential age distribution has been assumed. In the treated animals, cell velocity at the top of the crypt is 2·0 cell positions per hour, whereas in normals this value is 1·6/h. Thus we can conclude that the elongated abnormal crypts of the carcinogen-treated animals are producing a greater number of progeny than normal crypts. A comparison of the slope of the cumulative-birth-rate curves, however, shows that within the proliferative compartment the normal rises more steeply than the abnormal. Once again this effect appears to be a manifestation of a lower growth fraction within the proliferative compartment.

### DISCUSSION

### *Crypt hyperplasia*

These results show that during DMH treatment of rats crypt hyperplasia occurs in the proximal small bowel. There are apparently 2 separate phases: initially a sustained modest elongation is seen from control levels of about 34 cells to 36–40 cells; and after about 24 weeks of treatment a sudden, much more dramatic elongation to 43–63 cells is seen. This latter increase in crypt length coincides with the development, in an increasingly large proportion of animals, of foci of atypia and of frank malignant neoplasms largely localized to the region under study. This crypt hyperplasia is not explicable simply on the basis of ageing (Clarke, 1977), and it is tempting to speculate that it represents a preneoplastic phase.

Nevertheless, hyperplasia of small-intestinal crypts has been described in a variety of circumstances not obviously related to the development of neoplastic disease. Cairnie and Bentley (1967) described crypt hyperplasia as a normal physiological event during lactation. They found an increase in column count also, and a suggestion of increased migration rates; growth fraction remained unchanged.

As regards pathological states, gluten-sensitive enteropathy in man is characterized by crypt hyperplasia, associated with an increased rate of cell migration from the crypts (Wright *et al.*, 1973). It was considered on the basis of mitotic-index-distribution analysis that the growth fraction was slightly lower in coeliac patients than in controls, and there was good evidence of a shortening of the cell-cycle time in the proliferative compartment. Various workers have produced models of crypt hyperplasia in experimental animals. When rat jejunum is explanted to the skin surface a state of crypt hyperplastic villous atrophy results, with a greatly increased rate of migration of cells on to the surface of the mucosa (Loehry and Grace, 1974). Crypt hyperplasia has been described in rats infested with the nematode *Nippostrongylus brasiliensis* (Symons, 1965). Once again the migration rate of cells out of the crypts seemed to be enhanced. These conditions of crypt hyperplasia appear to represent a response to some irritant or inflammatory process

FIG. 6.—Examples of accumulation of mitosis after vincristine (Vcr.) administration: (a) Cell Position 1–4, (b) 9–12, (c) pooled whole crypt. The dotted lines indicate the 95% confidence limits for the fitted lines.

causing an increased rate of loss of mature cells from the mucosal surface.

Wiebecke *et al.* (1973) described "more or less localised mucosal hyperplasias" occurring in the small bowel of DMH-treated rats, and mentioned the presence of sharply demarcated zones of undifferentiated epithelium within the crypts. Mucosal hyperplasias have also been described in the colon of experimental animals during DMH carcinogenesis (Wiebecke *et al.*, 1973; Tutton and Barkla, 1976). In the latter authors' experience, increase in the circumference of the crypts was more prominent than elongation.

*Growth fraction*

From the form of the labelling-index distribution curves it is quite obvious that in the DMH-treated animals there is a considerable increase in the absolute size of the so-called proliferative compartment. However, the ratio of the site with 50% of the peak labelling to the total crypt height in fact falls from 0·59 in control animals to 0·56 in treated animals. This small change is probably not significant.

Considering the ratio of observed labelling index to theoretical labelling index (derived from FLM curves), we find an $I_p$ estimate for the whole crypt of about 0·49 in DMH-treated animals, which is substantially less than the value of 0·67 calculated using data from normal animals from the same colony. This we consider reliable evidence that the growth fraction falls in the hyperplastic crypts of DMH-treated animals. Furthermore there is the strong suggestion that much of this reduction in proliferating population is occurring within the so-called proliferative compartment, and may be due to the alkylating properties of metabolites of DMH (Pozharisski *et al.*, 1975). There is, however, no histological evidence of cell

FIG. 7.—Cumulative birth-rate curves: (a) normal animals, (b) DMH-treated group.

damage or death. It is of interest to note that Loehry and Grace (1974) suspected a similar type of change, on the basis of simple labelling studies in their model. Wright and colleagues (1973) showed a fall in $I_p$ in coeliac patients, while in lactating rats, Cairnie and Bentley (1967) thought $I_p$ to be unchanged.

*Cell proliferation*

The results of the FLM experiment show that actual cell-cycle times remain more or less unchanged in treated animals. The small standard errors generated by the Gilbert programme are, we feel, a considerable underestimate in this situation. In the vincristine experiment a similar prolongation in $T_{c(a)}$ is seen to that noted by Tutton and Barkla (1976) in the colonic crypts of DMH-treated rats; $T_{c(a)}$ at virtually all sites is somewhat prolonged, and this is explicable on the basis of the fall in $I_p$. Cell-cycle times have seldom been estimated in other mucosal hyperplasias: in Nippostrongylus infestation, however, Symons (1965) found a fairly convincing reduction in $T_c$ using an FLM method, and Wright *et al.* (1973) found suggestive evidence of a fall in $T_{c(a)}$ in human coeliac mucosa.

In all the hyperplasias described in the reports cited, an increase in cell production rate has been described or inferred. The results of the present work show a modest increase in the rate of movement of cells from the top of the crypt with a value of 2·0 cell positions/h in DMH-treated animals and 1·6 cell positions/h in controls.

This work was supported by a grant from the North of England Council of the Cancer Research Campaign. We would like to thank Miss E. Wark who typed the manuscript, and Mrs E. Wallace and Mrs M. Hughes who provided technical assistance.

REFERENCES

AL-DEWACHI, H. S., WRIGHT, N. A., APPLETON, D. R. & WATSON, A. J. (1974) The Cell Cycle Time in the Rat Jejunal Mucosa. *Cell Tissue Kinet.* **7**, 587.

AL-DEWACHI, H. S., WRIGHT, N. A., APPLETON, D. R. & WATSON, A. J. (1976) Studies on the Mechanism of Diurnal Variation of Proliferative Indices in the Small Bowel Mucosa of the Rat. *Cell Tissue Kinet.* **9**, 459.

CAIRNIE, A. B. & BENTLEY, J. (1967) Cell Proliferation Studies in the Intestinal Epithelium of the Rat. Hyperplasia during Lactation. *Expl Cell Res.*, **46**, 428.

CAIRNIE, A. B., LAMERTON, L. F. & STEEL, G. G. (1965) Cell Prolieration Studies in the Intestinal Epithelium of the Rat: I Determination of the Kinetic Parameters. *Expl Cell Res.*, **39**, 528.

CLARKE, R. M. (1977) The Effects of Age on Mucosal Morphology and Epithelial Cell Production in the Rat Small Intestine. *J. Anat.*, **123**, 805.

CLEAVER, J. E. (1967) Cell Renewal in Small Intestine. In *Thymidine Metabolism in Cell Kinetics* Amsterdam: North-Holland.

DESCHNER, E. E. (1974) Experimentally Induced Cancer of the Colon. *Cancer*, **34**, 824.

DRUCKREY, H., PREUSSMANN, R., MATZKIES, F. & IVANKOVIC, S. (1967) Selektive Erzeugung von Darmkrebs bei Ratten durch 1,2 Dimethylhydrazin. *Naturwissenschaften*, **54**, 285.

DRUCKREY, H. (1970) Production of Colonic Carcinoma by 1,2 Dialkyl Hydrazines and Azoxyalkones. In *Carcinoma of the Colon and Antecedent Epithelium*. Ed. W. J. Burdette. Thomas: Springfield.

GILBERT, C. W. (1972) The Labelled Mitosis Curve and the Estimation of the Parameters of the Cell Cycle. *Cell Tissue Kinet.* **5**, 53.

LIPKIN, M. (1974) Phase 1 and Phase 2 Proliferative Lesions of Colonic Epithelial Cells in Diseases Leading to Colonic Cancer. *Cancer*, **34**, 878.

LOEHRY, C. A. & GRACE, R. (1974) The Dynamic Structure of a flat Small Intestinal Mucosa Studied on the Explanted Rat Jejunum. *Gut*, **15**, 289.

MARTIN, M. S., MARTIN, F., MICHIELS, R., BASTIEN, H., JUSTRABO, E., BORDES, M., & VIRY, B. (1973) An Experimental Model for Cancer of the Colon and Rectum. *Digestion*, **8**, 22.

PEGG, A. E. & HAWKS, A. (1971) Increased Transfer RNA Methylase Activity in Tumours Induced in Mouse Colon by DMH. *Biochem. J.*, **122**, 121.

POZHARISSKI, K. M., KAPUSTIN, YU. M., LIKHACHEV, A. J. & SHAPOSHNIKOV, J. D. (1975) The Mechanism of Carcinogenic Action of 1,2 Dimethylhydrazine (SDMH) in Rats. *Int. J. Cancer*, **15**, 673.

REDDY, B. S., NARISAWA, T., WRIGHT, P., VUKUSICH, D., WEISBURGER, J. H. & WYNDER, E. L. (1975) Colon Carcinogenesis with Azoxymethane and Dimethylhydrazine in Germ-free Rats. *Cancer Res.*, **35**, 287.

SIGDESTAD, C. P., BAUMAN, J. & LESHER, S. (1969) Diurnal fluctuations in the Number of Cells in Mitosis and DNA Synthesis in the Jejunum of the Mouse. *Expl Cell Res.*, **58**, 159.

SYMONS, L. E. A. (1965) Kinetics of the Epithelial Cells and Morphology of Villi and Crypts in the Jejunum of the Rat Infected by the Nematode *Nippostrongylus brasiliensis. Gastroenterology*, **49**, 158.

TANNOCK, I. F. (1967) A Comparison of the Relative Efficiencies of Various Metaphase Arrest Agents. *Expl Cell Res.*, **47**, 345.

TUTTON, P. J. M. & BARKLA, D. H. (1976) Cell Proliferation in the Descending Colon of Dimethylhydrazine treated Rats and in Dimethylhydrazine induced Adenocarcinomata. *Virchows Archiv. B Cell Path.*, **21**, 147.

WIEBECKE, B., KREY, U., LÖHRS, U. & EDER, M. (1973) Morphological and Autoradiographical Investigations on Experimental Carcinogenesis and Polyp Development in the Intestinal Tract of Rats and Mice. *Virchows Arch. A. Path. Anat.*, **360**, 179.

WIEBECKE, B., LÖHRS, U., GIMMY, J. & EDER, M. (1969) Erzeugung von Darmtumoren bei Mausen durch 1,2-Dimethylhydrazin. *Z. ges. exp. Med.*, **149**, 277.

WRIGHT, N. A. (1971) Variation in Tritiated Thymidine Uptake during DNA Synthesis in the Adrenal Cortex. *Histochemie*, **28**, 99.

WRIGHT, N. A. (1974) M.D. Thesis, University of Newcastle upon Tyne.

WRIGHT, N. A., AL-DEWACHI, H. S., APPLETON, D. R. & WATSON, A. J. (1975) Cell Population Kinetics in the Rat Jejunal Crypt. *Cell Tissue Kinet.*, **8**, 361.

WRIGHT, N. A., MORLEY, A. R. & APPLETON, D. R. (1972) Variation in the Duration of Mitosis in the Crypts of Lieberkühn of the Rat; a Cytokinetic Study using Vincristine. *Cell Tissue Kinet.*, **5**, 351.

WRIGHT, N. A., WATSON, A. J., MORLEY, A. R., APPLETON, D. R. & MARKS, J. (1973) Cell Kinetics in Flat (Avillous) Mucosa of the Human Small Intestine. *Gut*, **14**, 701.